# Perceptions of the Future and Health Behavior in Adulthood

**DOI:** 10.1177/00914150241268018

**Published:** 2024-08-06

**Authors:** Mathias Allemand, Kyrsten C. Hill, Patrick L. Hill

**Affiliations:** 1 27217 University Research Priority Program “Dynamics of Healthy Aging”, University of Zurich, Zurich, Switzerland; 2 7548 Department of Psychological and Brain Sciences, Washington University in St. Louis, St. Louis, USA

**Keywords:** health behavior, future time perspective, opportunities and time, adulthood, bifactor models

## Abstract

Engagement in healthier lifestyle behaviors often is motivated by a focus on the future. However, there is limited research on the associations between health behavior and future time perspective, defined as people's tendency to perceive their future as expansive or as limited. Data came from a survey of U.S. adults (*N *= 805, 49.3% female; *M *= 50 years, range: 19 to 85 years). Participants completed measures of perceptions of future opportunities and time and health behavior. Opportunities and time factors were uniquely associated with health behavior. While the perceived opportunities factor was strongly associated with better health behavior, the time factor was associated with poorer health behavior. However, this latter association was dependent on individual demographic and health status differences. These findings suggest that perceptions of future opportunities can play an important role in health behavior engagement and thus represent an important target for health promotion.

Engagement in healthier lifestyle behaviors is often motivated by a focus on the future, in that individuals realize that actions taken now to promote their health will have consequences for their future wellbeing and development. Preliminary research has suggested an important role of perceptions of time remaining in the future in predicting health behavior (Adams & Nettle, 2009; Griva et al., 2015; Hall et al., 2015), although findings are inconsistent (see Kooij et al., 2018). One potential reason is that people differ in their perspectives of how much time and how many opportunities they have remaining. These perceptions, often referred to as future time perspective (Carstensen & Lang, 1996; Rohr et al., 2017), reflect a critical element of subjective aging, which has been consistently shown to be unique from chronological age (Allemand et al., 2022; Allemand & Hill, 2019). Past work has suggested at least two dimensions of future time perspective (Allemand & Hill, 2019; Zacher & Frese, 2009): perceptions of remaining time refer to the length of the personal time horizon, which is seen as extended or limited, and perception of remaining opportunities refers to the expansive or limited possibilities to pursue options, plans, and goals in the remaining lifetime.

The current study examined how FTP, using these two dimensions suggested by the past literature, may be linked to health behavior during adulthood. Healthy lifestyle behaviors may be encouraged by the belief one has more time and opportunities remaining, while individuals may be less motivated toward such behaviors if they perceive a more limited future. The current study investigated linkages between future time perspective and health behavior among adults, evaluating the robustness of these associations as well as whether they differ based on the form of perspective evaluated.

## Future Time Perspective and Adult Development

Multiple theories of adult development point to how chronological age provides at best a proxy for the aging process (e.g., Carstensen et al., 1999; Kotter-Grühn et al., 2015; Montepare, 2009), signifying the typical times when given life events and transitions may be more common. Moving beyond chronological age, these theories have consistently called for greater attention to people's subjective perceptions of the aging process. Efforts on this front have taken multiple forms, including assessing subjective age (Stephan et al., 2018) and perceived age across different domains (identity age, personal age, cognitive age; e.g., Barak, 1987; Settersten & Mayer, 1997). However, among the most frequently evaluated is future time perspective, given the rich theoretical foundation on which it rests.

Indeed, Socioemotional Selectivity Theory (SST; Carstensen, 2021; Carstensen et al., 1999; Löckenhoff & Carstensen, 2004) has provided researchers with clear expectations for how adults may act and what they may prioritize across the adult lifespan. This framework suggests that how one perceives their remaining time and opportunities will shape their social goals. Specifically, SST suggests that adults who envision an expansive, unlimited future are more likely to focus on developing new partners and connections, whereas adults perceiving a more limited time horizon emphasize contact with close social partners. Individuals with more limited time perspectives are motivated to avoid confrontation and negative social experiences, allowing them to better regulate their emotional wellbeing. Support for this framework comes from multiple cross-sectional studies (see Carstensen, 1995; 2021; Carstensen et al., 2003 for reviews), which demonstrate that social partner preferences differ when people expect a more limited versus expansive future that lies ahead.

Most work couched in SST has focused on social and emotional wellbeing for these reasons, pointing to how adults with a more expansive time perspective tend to report better psychological wellbeing compared to adults with a more limited perspective (Allemand et al., 2021; Hill & Allemand, 2022; Kooij et al., 2018). One's perception of their remaining opportunities also plays a role in decision-making for multiple life domains. For instance, research has shown that future time perspective is associated with financial planning for retirement (e.g., Jacobs-Lawson & Hershey, 2005; Mooney et al., 2021), insofar that adults who expect more time remaining have different financial goals relative to adults who may view a need to capitalize on what opportunities still remain. Similarly, one would expect FTP to impact health and healthcare decisions as well.

## Future Time Perspective and Health Behavior

That said, less work in adulthood has considered FTP in the context of health behavior relative to the social and wellbeing domains. One meta-analysis identified only 15 studies evaluating physical exercise and 30 with respect to substance use, with the vast majority of the work being conducted with adolescent samples (Kooij et al., 2018). This omission of adult samples is important because a significant association between time perspective and substance use was found in studies with adolescent samples, but not for those with adult samples. However, strong conclusions are difficult to make given only three studies fell in the latter camp.

Connections to past research are further complicated by the inconsistent measurement of time perspective. Kooji et al.'s (2018) meta-analysis suggests that FTP scales are used less in time perspective research than other measures of time, such as time foci (e.g., are you thinking about the past, present, or future) and future consequences of current actions (e.g., Strathman et al., 1994; Zimbardo & Boyd, 1999). Such measures, though developmentally appropriate earlier in the lifespan, may be less applicable later into adulthood (Rule et al., 2024).

In addition, by employing these inventories, researchers are unable to explicitly compare whether perceptions of the future in terms of remaining opportunities and remaining time uniquely matter for health behavior. Although perceived opportunities and time are highly correlated, past research has shown that these dimensions may differentially predict emotional wellbeing and health outcomes (Allemand et al., 2022; Hill & Allemand, 2022). In addition, disentangling associations for perceived opportunities and perceived time holds value for application, as it can help interventionists understand which component may yield better capacity for changing unhealthy lifestyle behaviors in adulthood.

## Present Study

The present study addressed three primary goals. First, we sought to add to the existing literature by further evaluating bivariate associations between FTP and health behavior, and do so by moving beyond single-item, or single-construct behavioral indices. We expected that having a more expansive time perspective in terms of perceived opportunities and time would correlate positively with health behavior, captured as a composite of healthcare actions, nutrition, exercise, and substance use (Hampson et al., 2019). Second, we considered whether these associations were similar for perceived opportunities versus perceived time by evaluating multivatiate associations between the two dimensions of FTP and health behavior. Toward this end, we conducted bifactor models (Eid et al., 2017), which allow researchers to disentangle whether future time or opportunities predict health behavior above and beyond simply considering them as a single latent FTP construct. Given the novelty of this innovative analytic approach, we refrained from making assumptions regarding whether one or both dimensions would uniquely predict health behavior. Third, we evaluated the robustness of the associations between the two dimensions of FTP and health behavior, by examining to which extent these associations hold when including chronological age, adults’ health status, gender, education, and income as covariates, all variables that influence the likelihood and capability of acting in a healthy manner.

## Methods

### Sample and Procedure

Data come from a larger study examining associations between sense of purpose, meaning in life, future time perspective, health, and health behavior in a US sample of adults. The sample consisted of 805 participants (48.7% male; 49.3% female). Participants’ age ranged from 19 to 85 years (*M *= 50.31; *SD *= 16.34). Of the participants, 12.2% reported having a high school diploma, 29.6% some post-high school training (e.g., some college, vocational degree), 38.5% a bachelor's degree, and 16.9% an advanced professional degree (e.g., Master's degree, MBA). With respect to participants’ employment status, 15.6% were not currently employed, 11.1% were employed part time, 43.7% were employed full time, 14.6% were retired, 1.7% were currently studying, 2.0% were homemakers, and 11.3% were self-employed. With respect to participants’ relationship status, 30.7% were single, 42.8% were married or in a committed relationship and living together, 6.1% were married or in a committed relationship and *not* living together, and 12.3% were divorced. A total of 79.8% of participants identified as White, 11.1% as Black or African American, 6.0% as Asian or Pacific Islander, and 5.4% as Hispanic or Latinx (participants could check multiple options).

The study protocol was approved by the Ethics Committee of the Washington University in St. Louis (IRB ID# 202304002; date of approval: 4/4/23). Data collection took place in May 2023 using the Prolific panel service. Prolific provides researchers with the opportunity to advertise their studies to a broad participant base, targeting individuals with certain characteristics. Participants receive compensation for their work in line with the “good” pay rates at Prolific, currently around $12/hour of work. The only inclusion criteria for the present study were that individuals were 18 + years of age and currently living in the United States. In addition, we made sure that we had a sufficient number of older adults.

### Measures

*Future time perspective.* We followed previous work (e.g., Allemand et al., 2021; Allemand & Hill, 2019; Zacher & Frese, 2009) and used three items from the Future Time Perspective Scale (FTPS; Carstensen & Lang, 1996) to measure perceived remaining opportunities (FTP-O; i.e., “Many opportunities await me in the future”, “I expect that I will set many new goals in the future”, and “My future is filled with possibilities”), and three items to measure perceived remaining time (FTP-T; i.e., “Most of my life lies ahead of me”, “My future seems infinite to me”, and “As I get older, I begin to experience time as limited” [reverse coded]). The items were responded on a 7-point Likert-type scale ranging from 1 (very untrue) to 7 (very true). Higher scores indicate that people view their future as expansive and with greater opportunities. The Cronbach's alpha reliability estimates were FTP-O: α = .92 and FTP-T: α = .77.

*Health behavior.* Participants completed the 16-item Good Health Practice scale (GHP; Hampson et al., 2019). The GHP is based on items from the Health Behavior Checklist (HBC; Vickers et al., 1990) and covers a broad range of health behaviors (e.g., “I exercise to stay healthy”, “I get enough sleep”, “I see a doctor for regular checkups”). The item responses were on a 5-point Likert-type scale ranging from 1 (not at all like me) to 5 (very much like me). Higher scores indicate a greater number of health behaviors. Research has shown that higher levels of health behavior as assessed with the GHP score are negatively associated with biomarkers of cardiovascular and metabolic risk factors (Hampson et al., 2019). The Cronbach's alpha reliability estimate was α = .86.

*Covariates*. Subjective health was measured with the item “In general, would you say your health is” and includes five response options ranging from 1 (excellent) to 5 (poor) (*M *= 2.72, *SD *= 1.02). In addition, we considered age, gender (1 = male, 2 = female), education and income as covariates. Education was assessed in terms of the highest level of education from 1 (did not complete high school) to 5 (advanced professional degree) (*M *= 3.57, *SD *= 0.96) and income from 1 (under $10,000) to 8 (over $150,000) (*M *= 4.05, *SD *= 1.76).

## Analytic Plan

The analyses were exploratory and not preregistered. We examined our research questions within a structural equation modeling (SEM) framework and performed two sets of analyses. First, we examined the bivariate associations between remaining opportunities and time and health behavior (Model 1). The three constructs were modeled as three correlated latent factors. We used three manifest item variables for remaining opportunities and three manifest item variables for remaining time. To model health behavior as a latent factor, we created parcels using the item-to-construct balance technique (Little et al., 2002) to form four manifest indicators rather than using single items as manifest indicators. To identify and scale the latent factor models, we set the loading of one manifest reference variable (i.e., the first item) to unity and the intercept of this reference variable to zero. Next, we added subjective health, age, gender, education, and income as manifest variables to the model and examined the associations with remaining opportunities and time and health behavior (Model 1a).

Second, we examined the multivariate associations between remaining opportunities and time and health behavior using (a) a SEM-based regression model (Model 2) and (b) two Bifactor S-1 models (i.e., general factor versus specific factor of opportunity and specific factor of time) (Models 3 and 4). Because the highly correlated subfactors of the FTP based on the scale might result in similar predictive findings across the factors, we also used a bifactor model in which the variance shared between all items (i.e., general factor) was separated from variance shared between subsets of items (i.e., specific factor). This would allow us to identify the unique contribution of subfactors beyond the general future time perspective factor. We used a S-1 bifactor model (Eid et al., 2017), in which one specific factor is removed to ensure the convergence of the model. The two factors were uncorrelated, so that the specific factor only represents the unique variance common to the negatively keyed items. To check for the robustness of the findings we controlled for potential effects of subjective health, age, gender, education, and income (Models 2a, 3a, and 4a).

All analyses were performed using maximum likelihood (ML) estimation with Mplus 8 (Muthén & Muthén, 1998–2017). To assess goodness of fit of the models, we examined the chi-square (χ^2^), comparative fit index (CFI), standardized root mean square residual (SRMR), and root mean square error of approximation (RMSEA) statistics. CFI values greater than .95, SRMR values less than .05, and RMSEA values less than .06 are typically considered to indicate that a SEM model is adequately parameterized, although values as low as .90 and as high as .10, respectively, are acceptable (Hu & Bentler, 1998). Based on commonly observed effect sizes in psychological research (Funder & Ozer, 2019), we interpret a correlation of *r *≥ .10 to be small, *r *≥ .20 to be moderate, and *r *≥ .30 to be strong. For all descriptions below, we employ the term “prediction” in the purely statistical sense, and do not make claims regarding causality or directionality, which is discussed after the results.

## Results

### Bivariate Latent Associations

We started with a three-factor SEM model that consists of perceived remaining opportunities (FTP-O), perceived remaining time (FTP-T), and health behavior as three correlated latent factors (Model 1). The model fit the data well (χ^2^(32) = 147.46, *p *< .001, CFI = .976, SRMR = .040, RMSEA = .067; 95% CI = .056; .078). The results indicated a strong correlation between FTP-O and FTP-T (*r *= .83, *p *< .001). Both FTP-O (*r *= .29, *p *< .001) and FTP-T (*r *= .14, *p *= .001) were significantly associated with health behavior, indicating that those participants who see more opportunities in the future and perceive the future as expansive report more health behavior.^1^

Next, we added all covariates (i.e., subjective health, age, gender, education, and income) as manifest variables to Model 1a and examined the bivariate correlations between the latent constructs and the manifest covariates. This resulted in an acceptable model fit (χ^2^(67) = 334.71, *p *< .001, CFI = .954, SRMR = .039, RMSEA = .070; 95% CI = .063; .078). Table 1 includes the estimated correlations between the latent variables and the manifest demographic variables. Both FTP-O and FTP-T were negatively associated with age, indicating that increasing age is associated with a more limited time perspective and less opportunities. Better subjective health was associated with a more expansive time perspective and more perceived opportunities. While education was unrelated to FTP-O and FTP-T, having a higher income was associated with a more expansive future time perspective and greater opportunities in the future. Subjectively healthy, older, female adults with a higher education and income reported higher health behavior scores.

**Table 1.** Estimated Correlations between Latent Variables of Remaining Opportunities, Time, and Health Behavior and Demographic Variables (Model 1a).

	1	2	3	4	5	6	7
1. Remaining opportunities	–						
2. Remaining time	.82***	–					
3. Health behavior	.29***	.12**	–				
4. Subjective health^1^	-.38***	-.32***	-.40***	–			
5. Age	-.21***	-.51***	.30***	.002	–		
6. Gender	-.07	-.18***	.15***	.07	.26***	–	
7. Education	.03	-.06	.39***	-.20***	.16***	.07	–
8. Income	.19***	.16***	.25***	-.21***	-.02	-.08*	.33***

*Note.* ^1^A high score in subjective health reflects poorer health status.

*p < .05, **p < .01, ***p < .001.

### Multivariate Latent Associations

**Figure 1.**
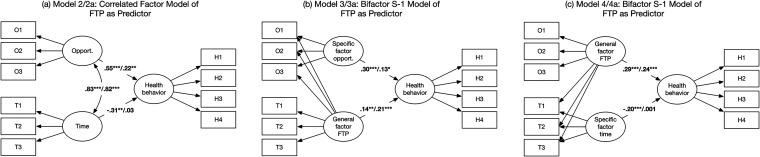
Correlated factor model (model 2) and bifactor S-1 models of future time perspective (models 3 and 4) as predictor of health behavior. *Note*. O1 to O3 represent remaining opportunities items (FTP-O: “I feel that many opportunities await me in the future”, “I expect that I will set many new goals in the future”, and “My future is filled with possibilities”); T1 to T3 represent remaining time items (FTP-T: “Most of my life lies ahead of me”, “My future seems infinite to me”, and “I begin to experience time as limited” [reverse coded]); H1 to H4 represent health behavior parcels (H1 parcel consists of the Good Health Practice scale (GHP) items 2, 10, 11, 13; H2 parcel consists of the GHP items 1, 7, 12, 15; H3 parcel consists of the GHP items 4, 5, 9, 16; H4 parcel consists of the GHP items 3, 6, 8, 14). General factor FTP includes variance shared between all items; specific factor opportunities/time includes variance shared between subsets of items to examine the uniqueness of the specific factor. Values represent standardized factor loadings or correlations. In Models 2a, 3a, and 4a, we controlled for subjective health, age, gender, education, income; for simplicity, we did not include the covariates in the figures. Values to the right of the slashes represent standardized factor loadings or correlations after controlling for the covariates. *p < .05, **p < .01, ***p < .001.

Second, we modeled a SEM-based regression model with the two correlated dimensions of FTP as predictors of health behavior (Model 2; see Figure 1 left). In terms of model fit, this model is equivalent to the three-factor SEM model (Model 1). FTP-O was positively related to health behavior (β = .55, *p *< .001), whereas FTP-T was negatively related to health behavior (β = -.31, *p *= .001). As noted above, however, the two dimensions of FTP as predictor variables were strongly correlated. Next, we added the covariates to this model (Model 2a), which has the equivalent model fit as Model 1a. FTP-O was still positively associated with health behavior (β = .22, *p *< .01), whereas FTP-T was not a statistically significant predictor anymore (β = .03, *p *= .761).

Due to the high correlation between the FTP subfactors, we next used a bifactor model in which the variance shared between all FTP-O and FTP-T items (i.e., general factor) was separated from variance shared between subsets of items (i.e., specific factor). First, we estimated a Bifactor S-1 model (Model 3; see Figure 1 middle) in which we modeled FTP-O as the specific factor (χ^2^(30) = 145.88, *p *< .001, CFI = .976, SRMR = .040, RMSEA = .069; 95% CI = .058; .081). The results indicate that both the general factor of FTP (β = .14, *p *= .001) and the specific factor of FTP-O (β = .30, *p *< .001) were associated with health behavior. That is, we found a unique contribution of opportunities beyond the general future time perspective factor. The inclusion of covariates (Model 3a) evidenced an acceptable model fit (χ^2^(65) = 327.05, *p *< .001, CFI = .954, SRMR = .039, RMSEA = .071; 95% CI = .063; .078). Controlling for covariates actually increased the predictive power of the general factor of FTP (β = .21, *p *< .001), but slightly decreased the predictive power of the specific factor of FTP-O (β = .13, *p *< .05) on health behavior. However, both predictors remained significant.

Next, we estimated a Bifactor S-1 model (Model 4; see Figure 1 right) with FTP-T as the specific factor (χ^2^(30) = 135.36, *p *< .001, CFI = .979, SRMR = .036, RMSEA = .066; 95% CI = .055; .078). The results indicate that the general factor of FTP (β = .29, *p *< .001) was positively and the specific factor of FTP-T (β = -.20, *p *< .001) was negatively associated with health behavior. Put differently, when accounting for the shared variance across FTP items, perceiving *less* time remaining was associated with better health behavior. The inclusion of covariates (Model 4a) evidenced an acceptable model fit (χ^2^(65) = 266.65, *p *< .001, CFI = .964, SRMR = .032, RMSEA = .062; 95% CI = .054; .070). The general factor of FTP (β = .24, *p *< .001) was still significantly associated with health behavior after the inclusion of the covariates, but the specific factor of FTP-T was no longer related to health behavior (β = .001, *p *= .977).

## Discussion

Past research has suggested a link between perceptions of future time and health behavior, although work has been limited to adult samples. The current study contributed to this literature in three important ways. First, the present findings provide further evidence that having an expansive time perspective is associated with a healthier lifestyle, in line with past research (e.g., Griva et al., 2015; Hall et al., 2015). It appears that adults with an expansive time perspective are more likely to recognize the link between immediate health promotion and better health in the future and, accordingly, are more likely to adopt a healthy lifestyle. Second, extending previous research (e.g., Kooij et al., 2018), we have shown that it is useful to conceptualize FTP as a multidimensional construct. Although the two dimensions of FTP are strongly related, the analyses argue for a distinction between perceived opportunities and time. The bivariate analyses indicate a strong association between perceived opportunities and health behavior, modeled as latent variables, whereas perceived time is moderately associated with health behavior.

Our multivariate findings further suggest that perceived opportunities and perceived time are uniquely associated with health behavior. Interestingly, analyses that simultaneously considered opportunities and time as predictors found that these constructs held different relations with health behavior. While the perceived opportunities factor was positively and strongly related to healthy lifestyle behaviors, the time factor was surprisingly *negatively* associated with health behavior. One possible explanation is that the two dimensions of FTP provide different motivations for health behavior. Extensive opportunities for the future motivate good health, but the limited time left to live can also motivate good health. In contrast, both limited opportunities and a long lifetime can make good health seem less urgent. Future research is needed to investigate these assumptions. That said, it is worth noting that this unexpected result could simply reflect a methodological artifact, as the two FTP dimensions are strongly related and therefore could have led to multicollinearity in the regression analysis. Therefore, we next employed bifactor models to better identify the unique contribution of perceived opportunities and perceived time beyond the general FTP factor. The bifactor models suggest that both opportunities and time provide significant predictive value for health behavior above and beyond a general FTP factor they are assumed to comprise. Specifically, perceived opportunities were positively associated and perceived time was negatively associated with health behavior.

The present findings provide further support for the multidimensional nature of FTP (Allemand et al., 2021; Hill & Allemand, 2022) and suggest the potential for these factors to differentially operate on health behavior. The unexpected negative relationship between future time perspective and health behavior suggests that perceiving future time as limited is associated with healthier behaviors, whereas an expansive future time perspective appears more detrimental to health behavior. Socioemotional Selectivity Theory (SST; Carstensen, 2021; Carstensen et al., 1999) states that perceptions of the future have important implications for the selection of goals, preferences, and activities. Specifically, when the future is viewed as limited or restricted, the focus shifts from the optimization of future possibilities to the maximization of meaningful activities and positive emotional experiences in the present. Based on this consideration, a limited time perspective could promote health behavior as a type of behavioral strategy to maintain positive emotional experiences. This result could also be related to the salience or immediacy of the consequences of health behavior. When people see their future as expansive, they may be less motivated to engage in health behavior because the consequences of doing nothing are further in the future and seem like a problem of the future they have time to address and correct later.

Finally, the current study contributed to this literature by showing that the positive unique association between perceived opportunities and health behavior is still robust but less strong when chronological age, adult subjective health status, gender, education, and income are taken into account as covariates. In contrast, the negative relationship between perceived time and health behavior disappears after controlling for multiple covariates that influence the likelihood and capability of acting in a healthy manner. Taken together, these results suggest that perceived opportunities may play a larger role in influencing health behavior than perceived time.

## Implications, Limitations, and Future Directions

Overall, our findings underscore the importance of considering perceived opportunities in efforts to influence engagement in healthy lifestyle behaviors. Perceptions of the future are modifiable and can therefore be targeted in interventions aimed at promoting health behavior. Previous work has developed potential routes for influencing perceptions of the future. For example, cognitive-behavioral interventions might be used to observe and reframe the way people attend to, think about, and recall information about their future (e.g., Beck, 2021). One key technique is “time projection” (e.g., Palmer & Cooper, 2007), which uses imagination to shift the perceptions of the future. This technique can be used to imagine a future full of opportunities and to mentally optimize the future by focusing on options, plans, and goals. Another technique called a “letter from the future” (Flower, 2021) helps people to explore, articulate, and build commitment towards a future goal or vision by engaging in writing. Both techniques can be adapted to promote more expansive perceptions of future opportunities, and future research should consider if they in turn can contribute to a healthy lifestyle.

There are a number of limitations in the present study that should be addressed in future work. First, given the cross-sectional nature of the present study, we cannot make claims regarding whether perceptions of the future affect health behavior or vice versa. Future research is needed to examine the directionality of associations through experimental manipulations of perceived opportunities. Another interesting direction is to examine the relationships between FTP and health behavior at the daily level using intensive longitudinal studies to capture how daily context either promotes or constrains perceptions of the future and health behavior activities. Second, the present research was based on self-reports, which leaves open the possibility for biased response styles and self-evaluation tendencies. However, it is important to note that perceptions of the future represent internal aspects of individual functioning; therefore, FTP levels are best captured via self-report. In contrast, the assessment of health behavior by self-reports can be supplemented by observer reports by close informants (e.g., relatives, friends) or objective measures of health behavior, including accelerometer or step counts to measure physical activity. Third, the present study focused on the associations between two dimensions of FTP and health behavior in a sample of adults with a broad age range, treating age as a covariate. However, work should consider whether associations between FTP and health behavior differ across adulthood. Although past work has found no evidence for age moderation for FTP associations with other constructs (Allemand et al., 2021; Allemand et al., 2022), future research is needed to examine potential moderating effects of age on the associations between FTP and health behavior.

## Conclusion

Although research has frequently considered the potential for adolescents’ views of their future to influence their health behavior (e.g., Johnson et al., 2014; Nurmi, 1991), the current study adds to the more modest literature with adult samples. Our findings support the need for future research on this topic, while also pointing to the potential equivocal nature for associations. Specifically, while perceptions of future opportunities hold consistent positive associations with health behavior, our work points to the potential for perceived remaining time to differ in its associations based on the analytic approach taken. Future research is needed to evaluate whether perceived remaining opportunities provide a pathway to improve health behavior, as well as when and how viewing one's time as limited could catalyze healthy activity among adults.

## Notes

1. We additionally tested a two-factor model consisting of a FTP factor (the FTP-O and FTP-T items loaded on only one latent factor) and a health behavior factor. However, this model resulted in a worse model fit (χ^2^(34) = 310.56, *p* < .001, CFI = .944, SRMR = .056, RMSEA = .101; 95% CI = .090; .111) (see also Allemand et al., 2021). The correlation between FTP and health behavior was *r* = .27, *p* < .001.
